# How to position the patient? A meta-analysis of positioning in vestibular schwannoma surgery *via* the retrosigmoid approach

**DOI:** 10.3389/fonc.2023.1106819

**Published:** 2023-02-01

**Authors:** Martin Vychopen, Felix Arlt, Erdem Güresir, Johannes Wach

**Affiliations:** Department of Neurosurgery, University Hospital Leipzig, Leipzig, Germany

**Keywords:** semi-sitting, lateral, positioning, vestibular schwannoma, venous air embolism, facial nerve, mortality

## Abstract

**Objective:**

Patient positioning is a matter of ongoing debate in the surgical treatment of vestibular schwannoma (VS). Main endpoints of this discussion are preservation of facial nerve functioning, extent of resection, and complications. In this meta-analysis, we aim to investigate the impact of patient positioning on VS surgery *via* the retrosigmoid approach.

**Methods:**

We searched for eligible comparative trials on PubMed, Cochrane library, and Web of Science. Positioning groups were compared regarding facial nerve outcome, extent of resection, postoperative hydrocephalus, postoperative CSF leaks, perioperative venous air embolism, and perioperative mortality. Two groups of positions were defined, and the following positions were allocated to those groups: (1) Semi-sitting and Sitting-position; (2) Lateral position, supine position with extensive head rotation, lateral oblique (=Fukushima/Three-quarter prone), and park-bench position.

**Results:**

From 374 full-text screenings, 7 studies met the criteria and were included in our meta-analysis comprising 1640 patients. Our results demonstrate a significantly better long-term (≥6 months) outcome of the facial nerve after VS surgery in the semi-sitting positioning (OR: 1.49, 95%CI: 1.03-2.15, *p* = 0.03). Positioning did not influence the extent of resection, rate of postoperative CSF leaks, and the presence of a postoperative hydrocephalus. Overall incidence of venous air embolisms was significantly associated with VS surgery in sitting positioning (OR: 6.77, 95% CI: 3.66-12.54, *p* < 0.00001). Perioperative mortality was equal among both positioning groups.

**Conclusion:**

Semi-sitting positioning seems to be associated with an improved facial nerve outcome after VS surgery *via* the retrosigmoid approach. Venous air embolisms are significantly more often observed among VS patients who underwent surgery in the sitting position, but the perioperative mortality is equal in both positioning groups. Both positioning groups are a safe procedure. Multicentric prospective randomized trials are needed to evaluate the risk-benefit ratio of each positioning in VS surgery *via* the retrosigmoid approach.

## Graphical Abstract

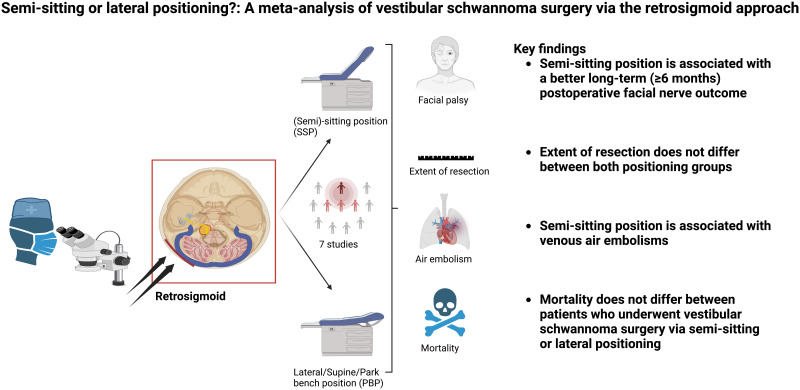

## 1 Introduction

Vestibular schwannoma (VS) is a benign neoplasm accounting for 75% of all tumors in the cerebellopontine angle, and it originates from the Schwann cells covering the vestibulocochlear nerve (1, 2). Gross total resection is suggested as the treatment of choice to achieve long-term tumor control (3). However, it is also of paramount importance to preserve the facial nerve. Furthermore, the perioperative mortality should be as low as possible in the context of a benign tumor disease. Due to the benign nature and slow growth rate, various therapy regimens have been introduced: watch and wait, radiotherapy or radiosurgery, and surgical resection (4, 5). The individual treatment of choice depends predominantly on the patient´s physical status, age at diagnosis, symptoms, and tumor size.

The retrosigmoid approach is the workhorse approach for enabling a microsurgical dissection in the Cerebellopontine angle. The positioning of VS patients during surgery *via* the retrosigmoid approach is highly debated. The lateral decubitus or supine positioning have been suggested for a long time as the benchmark positioning, whereas the semi-sitting positioning was underrepresented in most neurosurgical centers. The increased probability of a perioperative pulmonary air embolism and the preoperative logistical effort to perform a transesophageal echocardiogram in order to exclude a patent foramen ovale have been suggested as the major disadvantages of operating VS using the semi-sitting positioning (6). In contrast, recent monocentric comparative retrospective studies analyzing postoperative facial nerve functioning of VS patients who underwent either semi-sitting or lateral positioning found a potential superiority of the semi-sitting positioning regarding facial nerve outcome (7). To date, there is no high-level evidence (level 1 or 2) to support a general recommendation for of an individual preferred positioning for the retrosigmoid approach.

Against this backdrop, the present meta-analysis investigates comparative studies which analyzed lateral and semi-sitting positioning for VS surgery *via* the retrosigmoid approach. The primary aims of this meta-analysis are to identify the advantages and disadvantages of each kind of positioning regarding facial nerve outcome, incidence of pulmonary venous air embolism, extent of resection, and mortality.

## 2 Methods

For this systematic review we used the Cochrane Collaboration format (8) and the PRISMA checklist (9) were followed to conduct this systematic review.

### 2.1 Search strategy for identification of studies

We performed a systematic search of Pubmed database (http://www.ncbi.nlm.nih.gov/pubmed), Cochrane library, and Web of Science in September 2022. The search terms included *“*Vestibular Schwannoma*”* or *“*Acoustic Neuroma*”*. The search was limited to *“*human studies*”* and *“*English*”*. The inclusion criteria were formulated according to the PICOS (population, intervention, comparator, outcomes and study design) framework (10). These criteria were defined as follow: Subjects had undergone surgery for vestibular schwannoma; VS surgery was performed using the retrosigmoid approach; sitting/semi-sitting and lateral/lateral oblique/park-bench/supine positioning were compared; all results of the prespecified clinical endpoints are reported; and the studies were structured as comparative trials using those two defined positioning methods. The following kind of records were excluded: review articles, study protocols, conference abstracts, letters, unpublished manuscripts, animal experiments, and studies with insufficient data (e.g., clinical studies on the retrosigmoid approach in only one positioning group). All articles identified through the database search algorithm were evaluated for relevance according to a pre-defined scheme: First, title screening was performed to search for articles focusing on vestibular schwannoma. Subsequently, abstract screening, and, in case of further uncertainty, full-text screening was performed independently by two authors (MV and JV). Any disagreement between the reviewers concerning study inclusion or exclusion was resolved by consensus of a third author (E.G.).

### 2.2 Types of studies and types of positioning

We included all comparative clinical trials reporting on two different positionings of the patients for the resection of vestibular schwannoma *via* the retrosigmoid approach. After harvesting all the available data, we conducted the meta-analysis for following outcomes: facial nerve function according to House-Brackmann (11) dichotomized into good (≤2) and poor (>2), gross total/near-total resection rates, cerebrospinal fluid leak/hydrocephalus rates requiring medical therapy, air embolism and mortality. From an intraoperative hemodynamic and ventilatory point of view, lateral positioning, supine positioning, lateral oblique (=three-quarter prone, Fukushima) position, and park-bench position are very similar to each other (12). Those kind of positionings are suggested to have the benefit of a reduced risk of venous air embolisms, whereas the sitting position or the semi-sitting (= modified sitting positioning) position are suggested to reduce intraoperative bleedings. Therefore, two general types of positions were considered and the following positions were allocated to those groups: (1) (Semi)-Sitting positioning: Semi-sitting, and Sitting-position; (2) Lateral position, supine position with extensive head rotation, lateral oblique (=Fukushima/Three-quarter prone), and park-bench position.

### 2.3 Statistics

Review Manager Web (Revman Web Version 5.4.1 from the Cochrane Collaboration) was used to conduct the statistical analysis. We investigated the statistical heterogeneity by x^2^ and I^2^ statistics. An I^2^ value of 50% or more represented substantial heterogeneity. Weight of the relative contribution of the individual studies, based on the samples sizes, was included for the estimation of the treatment effects. Funnel plots were used to visually examine the publication bias of the included studies. Effect sizes were subsequently expressed as pooled odds ratio (OR) estimates in models using random effect.

## 3 Results

### 3.1 Literature search

**Figure 1:**
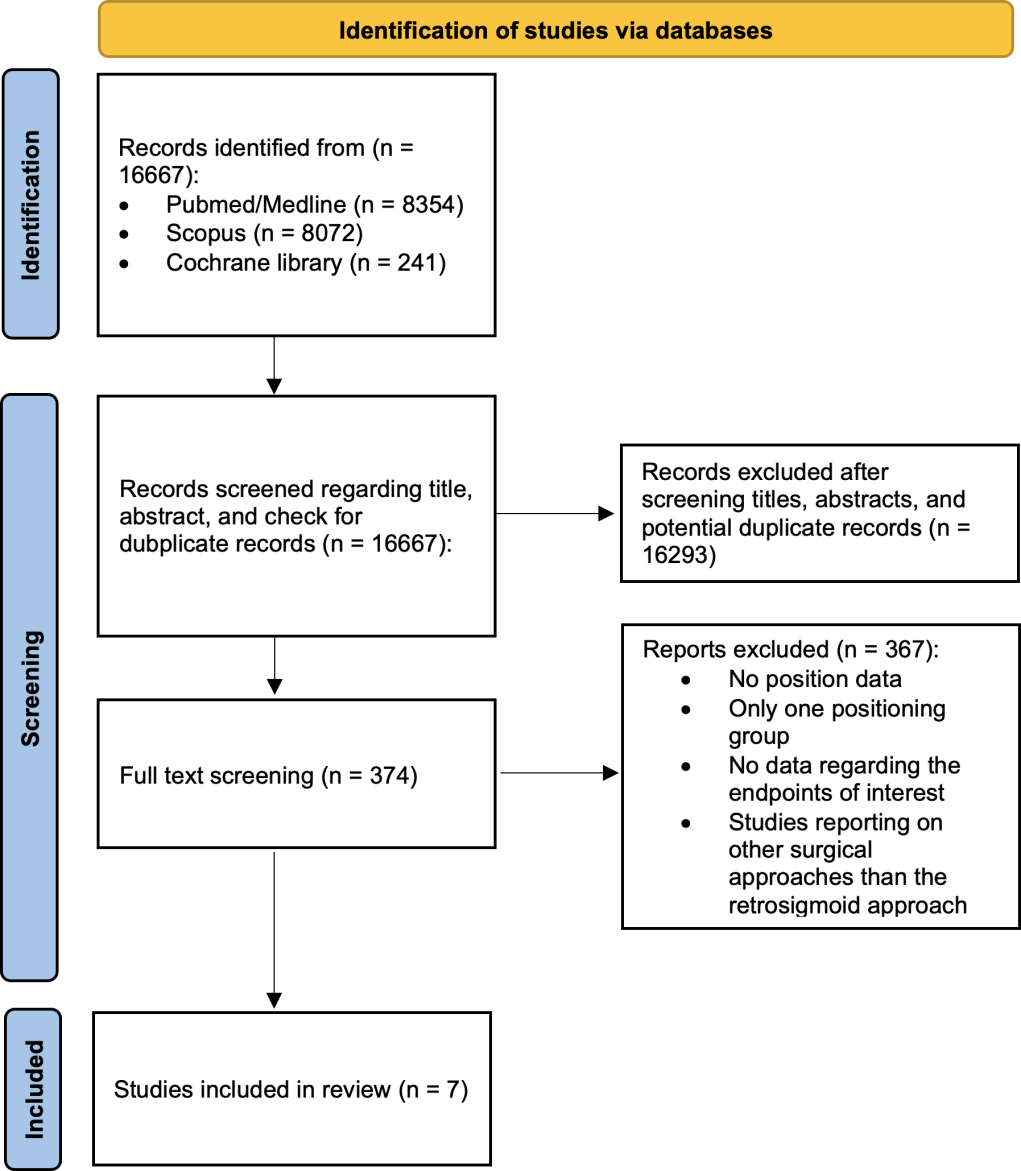
PRISMA flow chart illustrating the study selection of the present meta-analysis.

In total, 16667 English records were screened for possible eligibility. After title, abstract, duplicate records, and full-text screenings, 16660 records were excluded. Finally, 7 original articles involving 1640 patients met the inclusion criteria. **Figure 1** demonstrates the details of the literature search workflow.

### 3.2 Characteristics of included studies

The included studies were published from 1985 to 2022. **Table 1** summarizes the details of the patient positioning groups and the reported outcomes of all 7 included trials. All patients of the included studies underwent surgery for VS *via* the retrosigmoid approach either in the positioning group 1 or in the positioning group 2. There was no multi-arm (≥3 arms) trial among the included studies. Reported outcome data were allocated to the patient positioning groups (lateral & semi-sitting position) to conduct the pairwise meta-analysis of the dichotomous endpoints.

**Table 1:** Major characteristics of studies included in the present meta-analysis.

Name	Year	Study type	Types of postioing	Tumor size	Sample size (n)	Group 2: Lateral (n)	Group 1: Sitting/Semi-Sitting (n)	Good facial nerve outcome (n)	Gross total resection (n)	CSF leak (n)	Hydrocephalus (n)	Venous air embolism (n)	Mortality (n)	Country
**Song** (13)	2021	R	Semi-sitting and lateral	Diameters (median): 3.9 cm (group 1) vs. 3.2 cm (group 2)	259	156	103	81/125 69/89	125/156 89/101	3/156 4/101	0/156 1/101	0/156 2/103	0%	China
**Wach** (14)	2020	R	Semi-sitting and lateral	Tumor size classes (1/2/3 = 0-2, 2-4, >4 cm) (not stratified by positioning)	118	86	32	29/41 20/24	60/86 23/32	NA	9/86 1/32	0/86 0/32	1/86 0/32	Germany
**Schackert** (15)	2020	R	Semi-sitting and lateral	Koos classification: 1: 16 2: 104 3: 164 4: 229 (not stratified by positioning)	544	381	163	54/68 134/163	363/381 150/163	2/68 14/163	0/68 2/163	0/68 7/163	0/68 1/163	Germany
**Scheller** (16)	2019	P	Semi-sitting and supine	Koos classification in group 1: 1: 1 2: 21 3: 25. 4: 9 Koos classification in group 2: 1: 1 2: 13 3: 16. 4: 11	97	41	56	31/41 48/56	30/41 52/56	2/41 1/56	0%	0/41 2/56	NA	Germany
**Rössler** (7)	2016	R	Semi-siting and lateral	Diameters (mean): 20.7 mm (group 1) vs. 24.9 mm (group 2)	60	30	30	12/30 19/30	26/30 26/30	3/30 1/30	NA	0/30 1/30	0%	Germany
**Spektor** (17)	2015	R	Sitting and lateral	Diameters (median): 30.88 mm (group 1) vs. 29.20 mm (group 2)	130	80	50	NA	58/80 41/50	3/80 1/50	1/80 0/50	NA	0%	Israel
**Duke**(6)	1998	R	Sitting and supine	Diameters (mean): 2.8 cm (group 1) vs. 2.2 cm (group 2)	432	210	222	NA	NA	NA	NA	11/210 63/222	0%	USA

NA, Not available; P, Prospective; R, Retrospective.

### 3.3 Facial nerve

#### 3.3.1 General outcome

**Figure 2:**
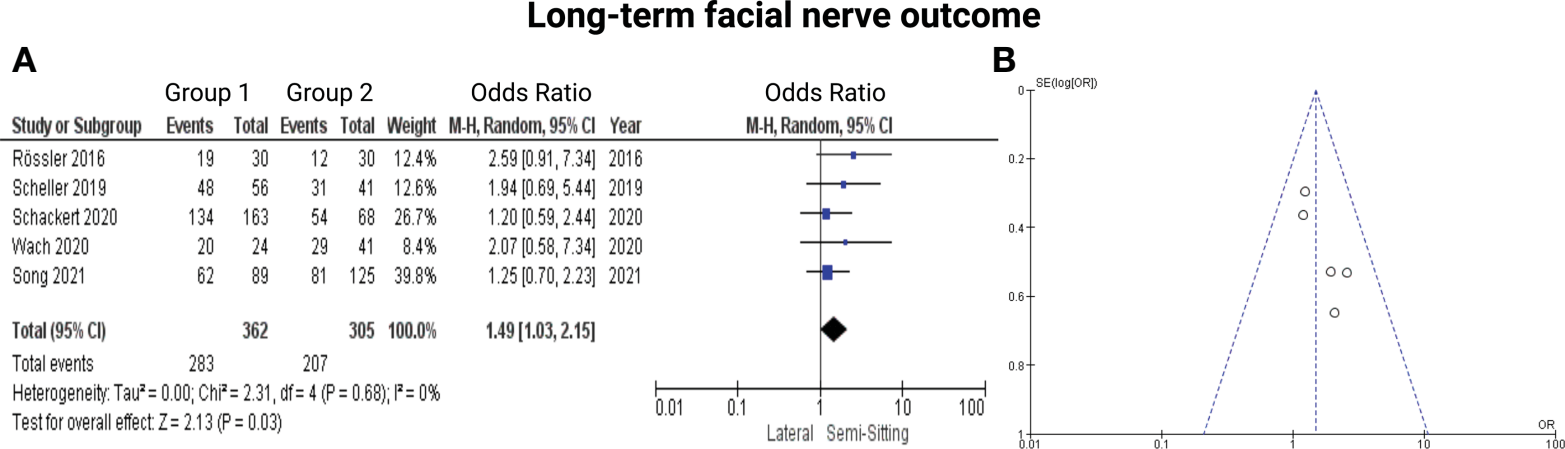
**(A)** Forrest Plots displaying OR and 95% CI estimates for facial nerve outcome comparison between Semi-Sitting and Lateral position and **(B)** Funnel plot showing no publication bias.

Five of the 7 included studies reported on postoperative function of the facial nerve (7, 13–16). Positioning group 1 exclusively included patients who underwent VS surgery in the semi-sitting positioning. Reported data allowed us to dichotomize the results according to House-Brackmann score (10) into good (≤2) and poor (>2). Altogether, 667 patients were allocated to either semi-sitting positioning (*n* = 362) or positioning group 2 (*n =* 305). In the positioning group 2, 207 out of 305 patients (67.8%) showed good postoperative facial nerve function compared to 283 out of 362 (78.1%) in semi-sitting position. Facial nerve outcomes were assessed either at 6- or at 12-months postoperatively. The odds ratio (OR) for good postoperative outcome in the pooled analysis was 1.49 (95% CI: 1.03 – 2.15, *p = 0.03*). I^2^ of 0% showed no significant heterogeneity among the studies (*p=0.68*). The Funnel-plot analysis showed no publication bias. For detailed information, see **Figure 2**.

Subsequently, we divided the studies according to the time of evaluation of the postoperative facial nerve outcome into 6-months outcome and 12-months outcome and analyzed the results accordingly:

#### 3.3.2 Outcome at 6-months after surgery for vestibular schwannoma

**Figure 3:**
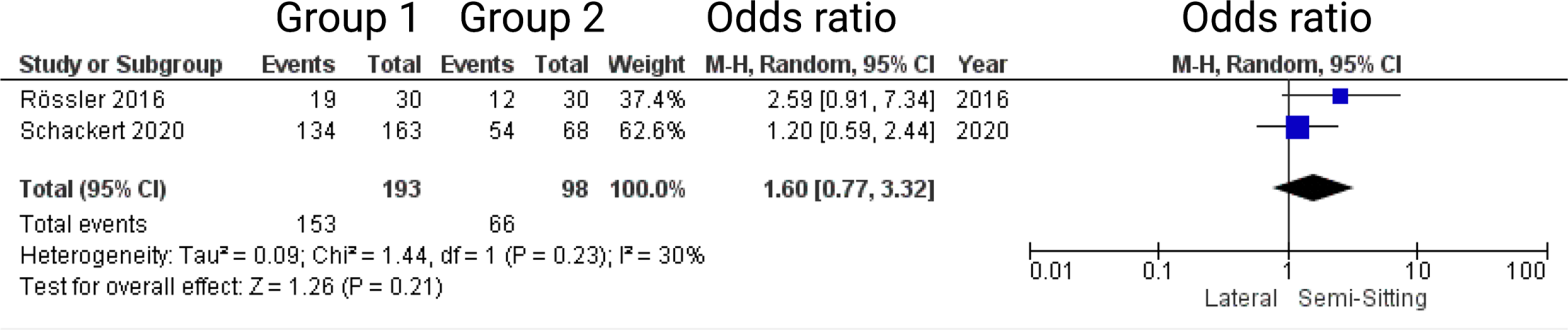
Forrest Plots displaying OR and 95% CI estimates for facial nerve outcome comparison between positioning group 1 (exclusively semi-sitting positioning) and positioning group 2 at 6 months postoperatively.

Two out of 7 studies report on facial nerve outcome at 6-months after surgery for VS (7, 15). Reported data allowed us to dichotomize the results according to House-Brackman score (10) into good (≤2) and poor (>2). Altogether, 291 patients were allocated to either semi-sitting positioning (*n* = 193) or positioning group 2 (*n =* 98). In positioning group 2, 66 out of 98 (67.3%) showed good outcome at 6-months postoperatively compared to 153 out of 193 patients (79.2%) in semi-sitting position. The odds ratio (OR) for good postoperative outcome in the pooled analysis was 1.60 (95% CI: 0.77 – 3.32, *p = 0.21*). I^2^ of 30% showed no significant heterogeneity among the studies (*p=0.23*). For detailed information, see **Figure 3**.

#### 3.3.3 Outcome at 12 months postoperatively

**Figure 4:**
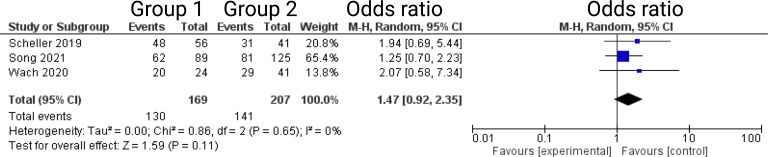
Forrest Plots displaying OR and 95% CI estimates for facial nerve outcome comparison between positioning group 1 (exclusively semi-sitting positioning) and positioning group 2 at 12 months postoperatively.

Three out of 7 studies reported on facial nerve outcome at 12-months after VS surgery (13, 14, 16). Reported data allowed us to dichotomize the results according to House-Brackman score (10) into good (≤2) and poor (>2). Altogether, 376 patients were allocated to either semi-sitting positioning (*n* = 207) or positioning group 2 (*n =* 169). In positioning group 2, 141 out of 207 patients (68.1%) showed good outcome 6-months postoperatively compared to 130 out of 169 (76.9%) in semi-sitting positioning group. The odds ratio (OR) for good postoperative outcome in the pooled analysis was 1.47 (95% CI: 0.92 – 2.35, *p = 0.11*). I^2^ of 0% showed no significant heterogeneity among the studies *(p=0.65)*. For detailed information, see **Figure 4**.

### 3.4 Extent of resection – Gross total resection

**Figure 5:**
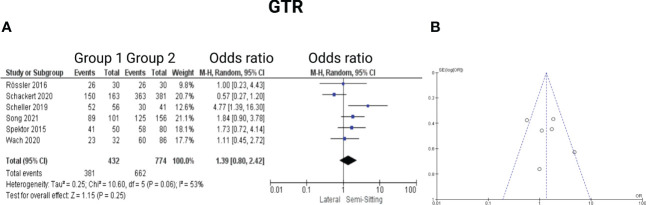
**(A)** Forrest Plots displaying OR and 95% CI estimates for gross total resection. **(B)** Funnel plot showing publication bias.

Six of the 7 included studies reported on the rates of gross total resection in surgery for VS (7, 13–17). Two studies further reported on the rates of near total resection (13, 16). Three studies reported on the frequencies of patients who did not underwent a gross total resection (7, 14, 15). Spektor et al. (17) reported data on patients who underwent a complete resection, subtotal resection, and partial resection, respectively. Reported data allowed us to dichotomize the results into gross totally and incompletely resected VS patients. Altogether, 1206 patients were allocated to either positioning group 1 (*n* = 432) or positioning group 2 (*n =* 774). In lateral position, 662 out of 774 patients (85.5%) underwent a gross total resection (GTR, whereas 381 out of 432 (88.2%) underwent GTR in sitting or semi-sitting position. The odds ratio (OR) for GTR in the pooled analysis was 1.39 (95% CI: 0.80 – 2.42, *p = 0.25*). I^2^ of 53% showed moderate, but not statistically significant heterogeneity among the studies *(p=0.06)*. For detailed information, see **Figure 5**.

### 3.5 CSF leak and postoperative hydrocephalus

#### 3.5.1 CSF leak

**Figure 6:**
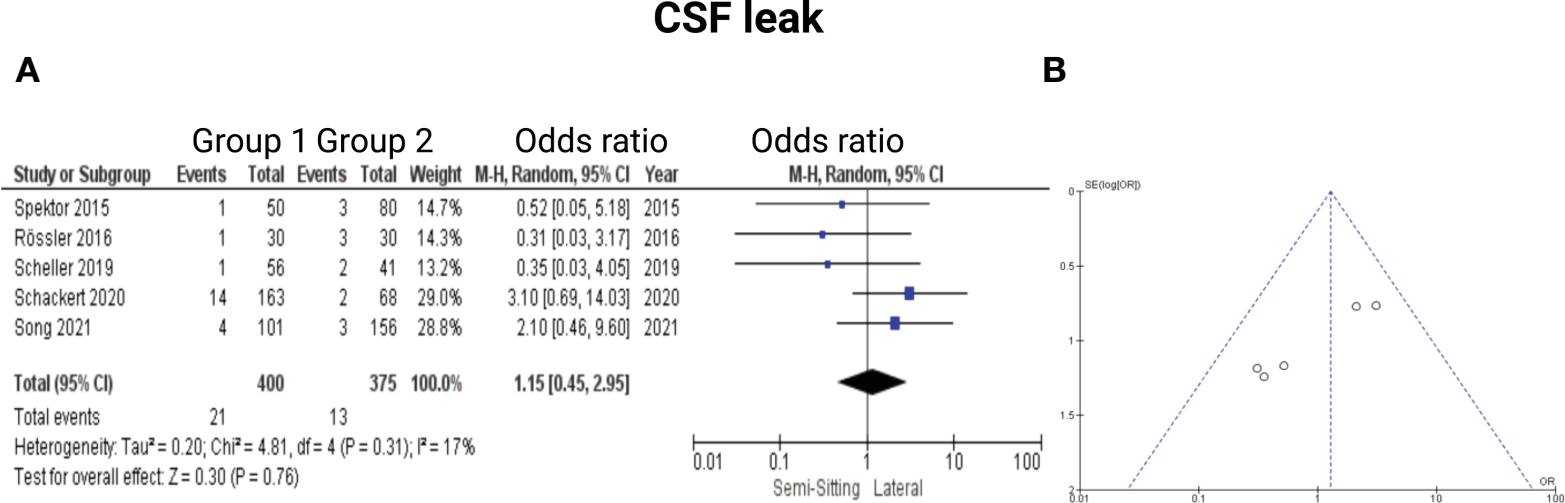
**(A)** Forrest Plots displaying OR and 95% CI estimates for CSF leak. **(B)** Funnel plot showing publication bias.

Five of the 7 included studies reported on CSF leak (7, 13, 15–17). Timepoints of the exact occurrence of postoperative CSF fistula were not given. All patients underwent a secondary treatment for CSF fistula (lumbar drain or revision surgery). Rössler et al. (7) stated that all CSF leak patients underwent a revision surgery. Reported data allowed us to dichotomize the results according to presence of CSF leak. Altogether, 775 patients were allocated to either positioning group 1 (*n* = 400) or positioning group 2 (*n =* 375). In positioning group 2, 13 out of 375 patients (4.9%) had CSF leaks compared to 21 out of 400 (5.3%) in sitting or semi-sitting position. The odds ratio (OR) for the presence of CSF leak in the pooled analysis was 1.15 (95% CI: 0.45 – 2.95, *p = 0.76*). I^2^ of 17% showed no significant heterogeneity among the studies *(p=0.31)*. For detailed information, see **Figure 6**.

#### 3.5.2 Postoperative hydrocephalus

**Figure 7:**
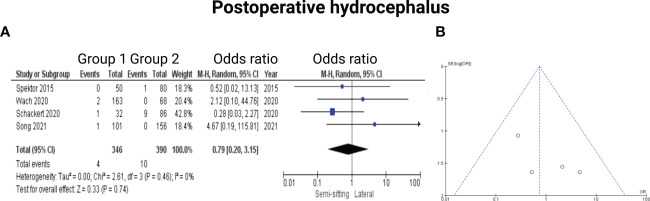
**(A)** Forrest Plots displaying OR and 95% CI estimates for Hydrocephalus. **(B)** Funnel plot showing publication bias.

Four of the 7 included studies reported on hydrocephalus (13–15, 17). Reported data allowed us to dichotomize the results according to presence of postoperative hydrocephalus. Altogether, 736 patients were allocated to either sitting or semi-sitting (*n* = 346) or positioning group 2 (*n =* 390). In positioning group 2, 10 out of 390 patients (2.6%) showed hydrocephalus compared to 4 out of 346 (1.6%) in the sitting or semi-sitting position. The odds ratio (OR) for the presence of hydrocephalus in the pooled analysis was 0.79 (95% CI: 0.20 – 3.15, *p = 0.74*). I^2^ of 0% showed no significant heterogeneity among the studies *(p=0.46)*. For detailed information, see **Figure 7**.

### 3.6 Venous air embolism

**Figure 8:**
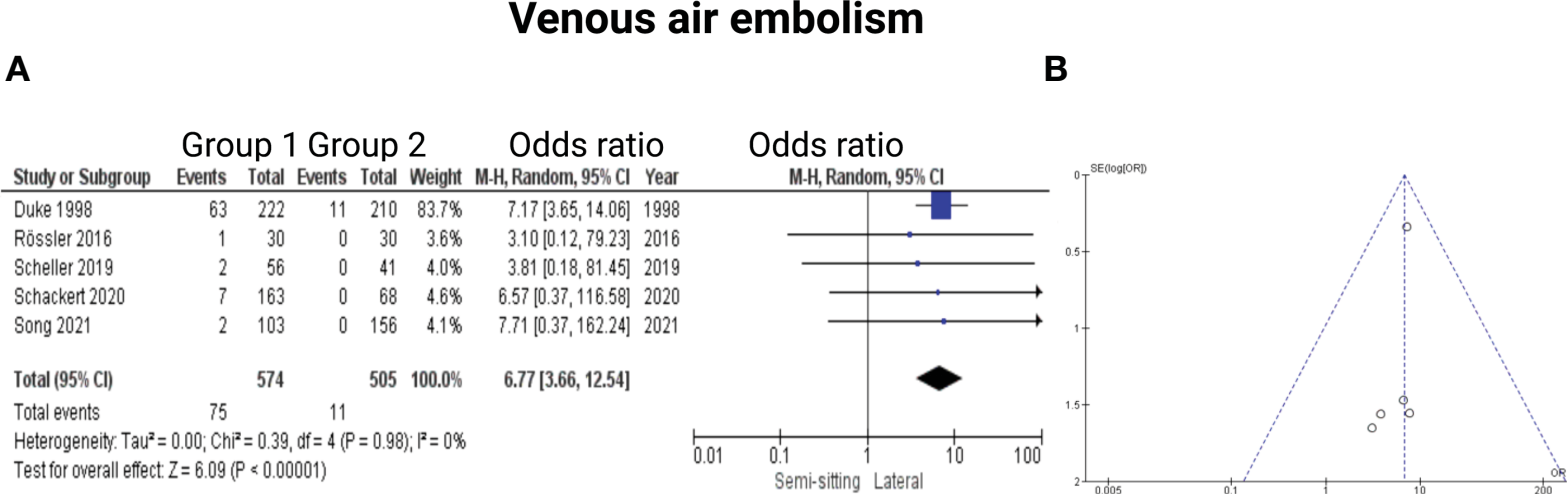
**(A)** Forrest Plots displaying OR and 95% CI estimates for venous air embolism. **(B)** Funnel plot for publication bias.

Five of the 7 included studies reported on venous air embolism (6, 7, 13, 15, 16). Reported data allowed us to dichotomize the results according to overall incidence of venous air embolism. Altogether, 1079 patients were allocated to either sitting/semi-sitting (*n* = 574) or positioning group 2 (*n =* 505). In positioning group 2, 11 out of 505 patients (2.2%) showed venous air embolism compared to 75 out of 574 (13.1%) in sitting or semi-sitting position. The odds ratio (OR) for the presence of venous air embolism in the pooled analysis was 6.77 (95% CI: 3.66 – 12.54, *p < 0.00001*). This effect was mainly based on the findings of the study by Duke et al. (6) in which patients of positioning group 1 underwent surgery in the sitting position. I^2^ of 0% showed no significant heterogeneity among the studies *(p=0.98)*. Funnel plot analysis showed no significant publication bias. For detailed information, see **Figure 8**.

### 3.7 Mortality

**Figure 9:**
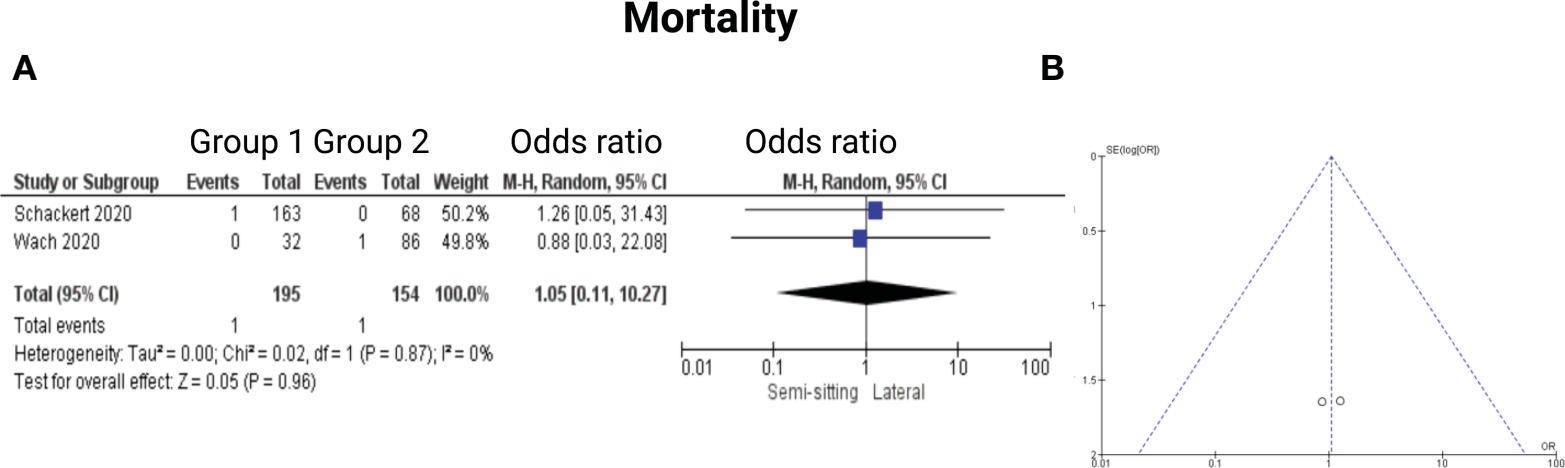
**(A)** Forrest Plots displaying OR and 95% CI estimates for mortality. **(B)** Funnel plot for publication bias.

Two of the 7 included studies reported on mortality (14, 15). Reported data allowed us to dichotomize the results. Altogether, 349 patients were allocated to either positioning group 1 (*n* = 195) or positioning group 2 (*n =* 154). In positioning group 2, 1 out of 154 patients (0.6%) deceased compared to 1 out of 194 (0.5%) in the sitting or semi-sitting position. The odds ratio (OR) for the mortality in the pooled analysis was 1.05 (CI 95%: 0.11 – 10.27), *p = 0.96*. I^2^ of 0% showed no significant heterogeneity among the studies *(p=0.87)*. For detailed information, see **Figure 9**.

## 4 Discussion

In the present meta-analysis, we have summarized the evidence from comparative studies investigating lateral and semi-sitting positioning for VS surgery using the retrosigmoid approach. Our main results can be summarized as follows: (1) The postoperative long-term (≥6 months) facial nerve outcome was significantly better in the patients who underwent VS surgery *via* the retrosigmoid approach in the semi-sitting position; (2) Extent of resection is not influenced by the patient positioning; (3) The rate of CSF leaks or hydrocephalus is equal among both positioning groups; (4) Pulmonary venous air embolism was significantly associated with sitting positioning; (5) Perioperative mortality is not influenced by the positioning and both methods are safe for the retrosigmoid approach.

Immediate worsening of the facial nerve functioning in the first weeks after surgery is a frequently observed dysfunction after VS surgery. Despite a preservation of the anatomical and electrophysiological continuity of the facial nerve, immediate worsening after surgery can often not be avoided. Predictors for postoperative facial nerve functioning are the extent of resection, neuropathological characteristics (e.g., MIB-1 index, macrophage density), intraoperative electrophysiological threshold and the proximal-to-distal amplitude ratio (14, 18–20). Postoperative 7^th^ nerve palsy can promote many serious side effects. Often, additional surgeries to treat facial nerve palsy induced comorbidities are necessary (e.g., hypoglossal-facial nerve anastomosis (21), treatment of eye and tear dysfunctions). To date, the retrosigmoid approach is the workhose for surgery of vestibular schwannomas in the cerebellopontine angle (22). Large tumors (classified according to Koos as T3 or T4) can be challenging and necessitate extensive surgical interventions (23). Facilitating surgery by placing the VS patients into the semi-sitting position reduces the use of surgical aspirators in the resection cavity by enhancing the venous drainage and irrigation fluid keeps the cavity clear during surgery. Hence, the surgeon might have an improved setting regarding the use of both hands and can work bimanually with a straight and direct view of the operating field at the stage of nerve structure preparation. The identification of the facial nerve can be very challenging and in giant VSs the facial nerve can be stretched around the schwannoma. Hence, a clear surgical view is of paramount importance to identify the nerve structures. The semi-sitting position might enable surgery with less bleeding, brain swelling, and reduced intracranial pressure due to the improved venous drainage of the cerebral blood flow (24). Furthermore, gravity enables an operating field free from blood and CSF (24). The continuous irrigation during semi-sitting surgery facilitates the bimanual dissection technique. Furthermore, Schackert et al. (14) showed that the intraoperative blood loss is significantly lower in patients who underwent VS surgery using the semi-sitting positioning. Hence, those favorable intraoperative physiological mechanisms in the semi-sitting VS patients seem to support a more ergonomic work of the neurosurgeon which reduces the factors resulting in surgical manipulations of the facial nerve.

We found no significant association between the patient positioning and the probability of a gross total resection in our meta-analysis. Extent of resection is of paramount importance regarding the long-term tumor control and patients who underwent a complete resection have a lower risk of recurrence (25). However, there is also evidence that subtotal resection with subsequent irradiation of tumor remnants is also a feasible method to provide a nearly equal result with regard to the probability of progression-free survival (25, 26). Nevertheless, a total tumor resection results in a 30-50% risk of a facial nerve dysfunction (27–31). Hence, there is a stringent need for each VS patient regarding the creation of a tailored treatment and follow-up schedule. Preserving cranial nerve functioning is the primary goal and precedes the goal of achieving maximal cytoreductive surgery.

The positioning of the VS patients in the retrosigmoid approach was not found to have a role in the frequency of postoperative CSF leaks. The incidence of CSF leaks after surgery for vestibular schwannoma *via* the retrosigmoid approach is reported with a broad range from 0% to 27% (32, 33). Hence, there is no optimum positioning to potentially reduce the patient’s risk of a secondary surgery to repair a CSF leak. Hydrocephalus is found in 3.7-42% of patients with a VS (34–37). Despite total tumor removal, hydrocephalus sometimes does not improve because of obstruction of the arachnoid granulations by proteins or hemorrhage (37, 38). In the present meta-analysis, we could not identify a role of the positioning regarding the development of a persistent hydrocephalus. Tumor size, increased age at diagnosis, and cystic tumor appearance are suggested as the main predictors of persistent postoperative hydrocephalus necessitating therapy (39).

Due to the inconsistency of reported data, we were not able to statistically examine the effect of the positioning on the surgery duration (see **supplementary Table 1**). The most consistent data was reported on skin-to-skin time. Schackert (15) and Roessler (7) show significantly shorter mean duration if surgery was performed in semi-sitting position. On the other hand, Spektor (17) favors lateral positioning with mean difference of 163.8 minutes. The possible explanation for these discrepancies might be the change of the surgical workflow over the course of the years demonstrated by Schackert et al. (15). In the analysis of surgery duration, Schackert shows the tendency towards skin-to-skin time reduction between 1991 and 2019, mainly for T3 and T4 tumors. Furthermore, semi-sitting positioning significantly reduced the skin-to-skin time in larger T3-4 tumors, whereas no influence on skin-to-skin time was observed in T 1-2 tumors (15).

These facts would theoretically support the sitting position for giant VSs because of the emerging availability of radiosurgical therapy for T1 and T2 VS. Most of the VS surgeries are indicated because of the mass effect on the brainstem and are caused by giant VSs of the T3-4 category, in which the semi-sitting position seems to significantly shorten the length of the procedure (15).”

The most feared adverse event in patients who underwent VS surgery *via* the retrosigmoid approach using sitting or semi-sitting positioning is venous air embolism. We found that semi-sitting positioning is significantly associated with an increased risk of venous air embolism. However, this finding has to be interpreted with caution because the severity of the venous air embolisms was not homogeneously classified (e.g., hemodynamic instability, length of stay in ICU) in the individual studies which did not allow a more detailed analysis. Venous air embolism can cause pulmonary edema, acute respiratory distress syndrome and acute right ventricular failure (40). The literature provides data with a wide range between 5.6-21% regarding the risk of venous air embolism (41–43). However, this wide range might be caused by the heterogeneous intraoperative monitoring techniques to detect a venous air embolism (e.g., transesophageal echocardiography, transthoracic doppler, capnography, mass spectrometry). For instance, it is known that a transesophageal echocardiography identifies significantly more venous air embolism events compared to a transthoracic doppler, but the incidence of clinically relevant venous air embolisms (drop in end-tidal carbon dioxide above 3 mmHg) using the transthoracic doppler is much higher (24). Hence, the majority of the venous air embolisms were found to be non-significant, and the patients had no clinically relevant sequelae (24, 44). This finding is also reaffirmed by our meta-analysis which found no association regarding the perioperative mortality and patient positioning in VS surgery *via* the retrosigmoid approach. Consequently, both methods seem to be safe and pulmonary venous air embolism in the semi-sitting positioning can be prevented and managed in an experienced interdisciplinary team. Indeed, a patent foramen ovale is considered to be an exclusion criterion for the semi-sitting position. In those cases, the patient should not underwent VS surgery in the semi-sitting positioning and they should be operated on in the prone, supine or lateral position. Nevertheless, the German Society of Anaesthesiology and Intensive Care approves semi-sitting positioning in neurosurgical patients if the benefits outrank the risks (45). The presence of a giant vestibular schwannoma with an increased risk of a new postoperative facial nerve palsy might be a potential indication for this tailored interdisciplinary teamwork using a semi-sitting positioning. The main strategy is to prevent venous air embolisms, and not the detection. Operating in the semi-sitting position necessitates a continuous communication and interdisciplinary teamwork with the neuroanesthesiologists. For instance, electrophysiological monitoring, intermittent bilateral jugular compression to identify venous leaks, moving the VS patients into “head down-feet up”, and hemodynamic management using continuous fluid administration to increase the central venous pressure are essential intraoperative steps which have to be communicated and decided interdisciplinary by the neurosurgeon and the neuroanesthesiologist. Against the background of equivalent perioperative mortality among semi-sitting or lateral positioning in VS surgery *via* the retrosigmoid approach, semi-sitting positioning should be strongly considered in the setting of an experienced interdisciplinary team if it is preoperatively known that the patients have no patent foramen ovale.

The major limitation of our meta-analysis of comparative trials investigating semi-sitting and lateral positioning is the retrospective design of the included studies. Moreover, we could not include the duration of the surgery in a reliable statistical analysis because of relevant differences regarding the definition of duration as well as the reported outcome data (e.g., median, mean values, lack of standard deviation). Furthermore, there are heterogeneous definitions of the venous air embolisms. Furthermore, six of seven studies reported the rates of complete VS resection but further analysis of the rates of near total resection was limited to only two studies. Moreover, the tumor size might be a potential confounder in the analysis of the facial nerve outcome. Heterogeneous measurements and definitions of tumor size did not allow an inclusion of this variable in our meta-analysis approach. However, the meta-analysis showed that the perioperative safety regarding mortality is equal among both positioning methods and the facial nerve might have a better outcome in the semi-sitting group. An ongoing prospective randomized controlled trial comparing semi-sitting and lateral position in vestibular schwannoma surgery might give some further essential insights into this interesting debate (Chinese Clinical Trial Registry: ChiCTR1900027550) (46).

## 5 Conclusion

Semi-sitting positioning seems to be associated with an improved facial nerve outcome after VS surgery *via* the retrosigmoid approach. The incidence of venous air embolisms is significantly higher among the patients who underwent VS surgery in the semi-sitting position, but there is no difference regarding perioperative mortality between semi-sitting or lateral positioning. Further multicentric prospective randomized trials with a homogeneous intraoperative neuroanesthesiological monitoring setup are needed to provide a detailed assessment of the risk and benefits of each positioning in the retrosigmoid approach.

## Author contributions

Data acquisition was performed by JW; MV, and EG performed the data interpretation. Writing and creation of figures were performed by JW, MV, and EG Proof reading was done by FA, and EG. All authors contributed to the article and approved the submitted version
