# Supplementary material for: How to position the patient? A meta-analysis of positioning in vestibular schwannoma surgery via the retrosigmoid approach
Source: Front Oncol. 2023 Feb 1;13:1106819. doi: 10.3389/fonc.2023.1106819 (PMC9929142; doi:10.3389/fonc.2023.1106819)
Supplement: Supplementary file 1 [file Table_1.docx]

| Supplementary Table 1: Duration of surgery | | | | | |
| --- | --- | --- | --- | --- | --- |
| Study name | Art of measurement | Statistics | Semi-sitting/Sitting (min) | Lateral/Supine | p value |
| Song 2021  (17) | Unclear | Median | 440 min (IQR 362.0, 534.5) | 402.5 min (IQR 343.5, 480.0) | 0.008 |
| Wach 2020  (13) | N/A | N/A | N/A | N/A | N/A |
| Schackert 2020  (14) | Skin-to-skin | Mean | 295 min | 348 min | <0.001 |
| Scheller 2019  (16) | Unclear | Mean | 239 min | 325 min | <0.0001 |
| Roessler 2016  (7) | Skin-to-skin | Mean | 183 min | 365 min | <0.0001 |
| Spektor 2015 (15) | Skin-to-skin | Mean ± SD  Median | 595.3 ± 202.4  595.3( IQR 285-1000) | 431.5 ± 129.7  431.6 (IQR 120-900) | <0.0001 |
| Duke 1998  (6) | Anesthesia duration | Mean | 356 | 340 | 0.14 |
| Abbreviations: IQR – interquartile range, N/A – not available, SD – standard deviation | | | | | |
